# *Cryptosporidium* oocyst persistence in agricultural streams –a mobile-immobile model framework assessment

**DOI:** 10.1038/s41598-018-22784-x

**Published:** 2018-03-15

**Authors:** J. D. Drummond, F. Boano, E. R. Atwill, X. Li, T. Harter, A. I. Packman

**Affiliations:** 1 Integrative Freshwater Ecology Group, Centre for Advanced Studies of Blanes (CEAB-CSIC), Blanes, Girona, Spain; 2 Division of Hydrologic Sciences, Desert Research Institute, Reno, Nevada USA; 3 Department of Environment, Land and Infrastructure Engineering, Politecnico di Torino, Torino, Italy; 4 Department of Population Health and Reproduction, University of California, Davis, California, USA; 5 Department of Land, Air, and Water Resources, University of California, Davis, California, USA; 6 Department of Civil and Environmental Engineering, Northwestern University, Evanston, Illinois USA

## Abstract

Rivers are a means of rapid and long-distance transmission of pathogenic microorganisms from upstream terrestrial sources. Pathogens enter streams and rivers via overland flow, shallow groundwater discharge, and direct inputs. Of concern is the protozoal parasite, *Cryptosporidium*, which can remain infective for weeks to months under cool and moist conditions, with the infectious stage (oocysts) largely resistant to chlorination. We applied a mobile-immobile model framework to assess *Cryptosporidium* transport and retention in streams, that also accounts for inactivation. The model is applied to California’s Central Valley where *Cryptosporidium* exposure can be at higher risk due to agricultural and wildlife nonpoint sources. The results demonstrate that hyporheic exchange is an important process to include in models characterizing pathogen dynamics in streams, delaying downstream transmission and allowing for immobilization processes, such as reversible filtration in the sediments, to occur. Although in-stream concentrations decrease relatively quickly (within hours), pathogen accumulation of up to 66% of the inputs due to immobilization processes in the sediments and slower moving surface water could result in long retention times (months to years). The model appropriately estimates baseflow pathogen accumulation and can help predict the potential loads of resuspended pathogens in response to a storm event.

## Introduction

Waterborne zoonotic pathogens pose a public health risk due to their consistent point and non-point sources, which can significantly impair the ecological quality of aquatic systems. Pathogens enter streams and rivers in a variety of processes including overland flow and groundwater filtration^1^. Rivers are a means of rapid and long-distance transmission of pathogenic microorganisms. Viruses, bacteria, and parasites persist for varying amounts of time, especially within streambed sediments^2,3^, leading to long-term disease transmission. Of particular concern is the protozoal parasite, *Cryptosporidium*, which can remain infective for weeks to months under cool and moist conditions, with the infectious state (oocysts) largely resistant to chlorination. The 50% infectious dose (ID_50_) for livestock-derived *Cryptosporidium*, specifically *C. parvum*, for healthy humans ranges between 10–1,000 oocysts^4^. Monitoring programs assess the microbiological quality of waters to minimize health risk associated with pathogenic microorganisms. However, as it is still unfeasible to experimentally monitor pathogen levels at the high spatiotemporal resolution often needed to assess risk, sampling is often complemented with a model^5^. Both environmental and hydrological processes control the residence time and persistence of pathogens within a stream network. Current models consider stream flow conditions, but it is imperative to incorporate the wide variety of processes that control the transport and retention of *Cryptosporidium* in a dynamic stream environment.

Commonly, hyporheic exchange – the two-way exchange of water with the underlying sediments induced by pressure variations associated with streamflow over stream channel topography – is ignored in surface water modeling of pathogen and microbial transport in streams^5–8^. However, as the size (5 μm) and specific gravity (1.05 g/cm^3^) of *Cryptosporidium* are low, it is mainly removed from the water column by hyporheic exchange^9–11^ and to a lesser extent by sedimentation via association with larger and denser suspended aggregates^12^. Microbial interaction with the streambed and other stream transient storage areas has been greatly underestimated by only assuming gravitational settling (with or without association with larger suspended aggregates) without considering the key mechanism of hyporheic exchange. Hyporheic exchange of particles differs from solutes because of strong particle deposition during porewater transport. Even though the settling velocities of fine particles are low, gravitational settling can be more important within porewaters, where porewater velocities are extremely small^10,11^. Filtration in the bed leads to pathogen immobilization^10,11,13^. However, filtered microbes and fine sediment are often remobilized, corresponding to reversible filtration^14–16^. This slow release of microbes after initial deposition has been observed in streams^17–19^. If models do not consider hyporheic exchange within the hyporheic region then pathogens will not be conceptualized as being in the nearbed or hyporheic region to experience these additional immobilization processes, thus underestimating pathogen immobilization and retention in streams. This underestimation of pathogen accumulation during baseflow can lead to inaccurate predictions for pathogen resuspension during storm events, when the majority of pathogens are transmitted downstream^20–23^.

The main objectives of this modeling study was to improve storm flow predictions of potential resuspended *Cryptosporidium* oocysts by (1) appropriately characterizing the transport and retention of *Cryptosporidium* during baseflow conditions through incorporating hyporheic exchange and immobilization processes, (2) calculating residence times of *Cryptosporidium* in surface water and accumulation in immobile zones, such as streambed sediments, to estimate long-term persistence, and (3) estimating *Cryptosporidium* accumulation during baseflow conditions that can potentially be resuspended during a storm event. We apply a previously developed mobile-immobile model for microbes^9,19^ to *Cryptosporidium*, which accounts for hyporheic exchange and transport through pore water, reversible filtration within the streambed, and inactivation of microbes, to accurately predict the long-term persistence of pathogenic microorganisms within stream storage areas. The mobile-immobile model framework is convenient for river transport as the water column can be considered mobile and material retained in streambed sediments or slow-moving surface waters is comparatively immobile^9,24,25^. This model framework, in contrast to previous work, was developed for microbial transport in streams to incorporate detailed measurements of transport and retention processes at multiple scales^9,18,19^, which allows the use of lab-scale measurements to parameterize key transport and retention processes and apply them to reach-scale modeling. We used the model to assess the transport, retention, and inactivation of *Cryptosporidium* within stream environments, specifically under representative conditions of California’s Central Valley, where pathogen exposure can be at higher risk due to agricultural and wildlife nonpoint sources. Comparison of modeling results with and without immobilization processes (i.e., reversible filtration within the streambed) provided novel insights into the significance of hyporheic exchange and subsequent immobilization processes on pathogen retention and long-term persistence within streams. This study provides new understanding of pathogen transport and retention dynamics in streams to help improve future risk assessment.

## Methods

### Study site

**Figure 1:**
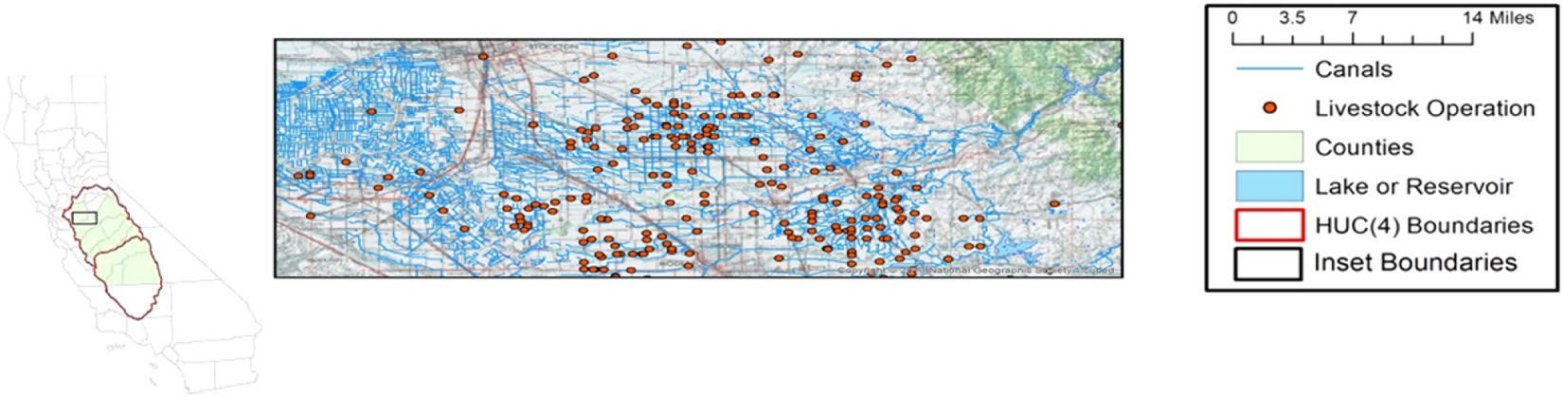
Central Valley and western slopes of the Sierra Nevada mountains. Rangeland animal agriculture is prevalent in the uplands, while dairy feedlots are prevalent in the valley.

The study site is California’s Central Valley and the Sierra Nevada foothills (Fig. 1), draining ~23,000 sq. miles of the western slope of the Sierra Nevada mountains down to the floor of the Central Valley. These streams originate in snow-fed lakes and streams surrounded by United States Forest Service and National Park lands where cattle and other livestock are grazed and descend through rolling foothills to the low-lying Central Valley, with foodcrop agriculture and animal feeding operations supported by a network of man-made canals. This site thus has potential inputs of fecal organisms from grazed allotments in the higher elevations and in animal feeding operations along the lower reaches and canals, in addition to inputs from wildlife such as striped skunks, coyotes, California ground squirrels, and yellow-bellied marmots^26,27^.

California’s Central Valley has a Mediterranean seasonal climate, with highly seasonal water inputs, with precipitation primarily in the winter and spring (December-May)^28^. Following the spring snowmelt period, most of the Central Valley is relatively dry with occasional summertime precipitation at higher elevations.

### Cryptosporidium inputs within study region

Approximately 1.7 × 10^5^ *Cryptosporidium* oocysts/animal/day are shed from a California adult beef cow and 6 × 10^5^ oocysts/animal/day from California beef calf^27,29^. An average beef herd size is approximately 100 adult cows and 150 calves, which equates to 1.1 × 10^8^ oocysts/day/livestock operation^30,31^. *Cryptosporidium* species include *C. bovis, C. ryanae*, and the more infectious *C. parvum*^32^. Oocysts deposited on the terrestrial portion of a watershed from beef cattle only reach streams through overland flow runoff and direct deposition of feces^27,33^. Oocysts remain trapped in the fecal matrix or are eluted and reach streams via groundwater or overland flow, with loads dependent on storm intensity, soil structure and infiltration rates^34,35^. The high risk fecal pats are those deposited directly into the stream, less than 1–5% of total fecal loads, depending on cattle access to the stream via lack of fencing. Thus, a reasonable estimate of *Cryptosporidium* that could potentially reach a stream in the region, estimating that 20 beef lots may impact a single stream, from a combination of direct deposition, overland flow, and groundwater inputs is approximately 1–5% of the oocysts shed. Dairy lots are assumed to have minimal release of *Cryptosporidium* oocysts as the operations are confined and runoff into stream is not permitted. Other non-point sources include wildlife, where the highest loads come from the California ground squirrel (*Spermophilus beecheyi*), averaging 1.13 × 10^5^ *C. parvum* oocysts/animal/day. Ground squirrel populations result in loading rates of 9 × 10^5^ oocysts/hectare/day for low density populations with 8 to 94 adults/hectare in California^29^. Therefore, non-point sources could potentially be a large source of *Cryptosporidium* to streams, even larger than beef herds and dairy lots combined. To account for these variable inputs, the highest figure (i.e., 5%) from beef herds was assumed to represent an upper boundary of the oocysts load, given the uncertainty in the number of oocysts shed by beef lots, the limited oocysts that may be released from dairy lots, and the additional contribution of non-point sources in the watershed from wildlife excretions.

## Model

### Mobile-immobile model framework for pathogen transport in streams

We modeled pathogen transport using a mobile-immobile model that can represent the effects of long residence time distributions^25^. This model has been previously applied to solute, fine particle, and microbial transport in rivers^9,18,19,36^ and is a modified version of the publicly available CTRW toolbox^37^. The mobile-immobile model is governed by advection and dispersion processes convolved with a memory function to represent storage in the system^25^:

1 $$\frac{\partial C(x,\,t)}{\partial t}={\int }_{0}^{t}M(t-t^{\prime} )[-v\frac{\partial C(x,\,t^{\prime} )}{\partial x}+D\frac{{\partial }^{2}C(x,\,t^{\prime} )}{\partial {x}^{2}}]dt^{\prime} $$

where *C* [M L^−3^] is in-stream concentration, *t* [T] is the elapsed time, *t’* [T] is a dummy time variable, *x* is downstream distance [L], *M*(*t*) [T^−1^] is the memory function (discussed below), and *v* [L T^−1^] and *D* [L^2^ T^−1^] are the velocity and dispersion coefficient in the stream. The memory function in Eq. 2 represents the mass or number of particles immobilized at time *t* that remain immobile at a later time (*t* + *dt*). Large variations in velocity, transient storage, and long-term particle retention can all be accounted for within the memory function. The Laplace transform, $\tilde{f}$(*u*), of a function *f* (*t*) is equal to ${\int }_{0}^{\infty }{e}^{-ut}f(t)dt$. The Laplace transform of the memory function *M(t)* is:

2 $$\tilde{M}(u)=u\bar{t}\frac{\tilde{\psi }(u)}{1-\tilde{\psi }(u)}$$

where *u* [T^−1^] is the Laplace variable and $\bar{t}$ is the average travel time in the reach, defined as the stream reach length divided by the mean water velocity (*v*). Equations (1) and (2) can be used to represent transport of pathogen transport for pre-specified residence time distributions. The overall residence time distribution in the stream $(\psi \,[{{\rm{T}}}^{-1}])$ is defined by the residence time distribution in the mobile region (water column) $({{\rm{\psi }}}_{0}\,[{{\rm{T}}}^{-1}])$, the rate of exchange from the water column to the immobile region (*Λ* [T^−1^]), and the residence time distribution in the immobile region (${\phi }_{i}\,[{{\rm{T}}}^{-1}]\,$), that will be defined for two different scenarios *i* = 1 and 2.

In Laplace space:

3 $$\tilde{\psi }(u)={\tilde{\psi }}_{0}[u+{\Lambda }-{\Lambda }{\tilde{\phi }}_{i}(u)]$$

Here, we assume that a single distribution ${\psi }_{0}$ characterizes the transport of solutes and pathogenic microorganisms by the surface flow, since these materials should be transported very similarly in the water column. We take this as an exponential distribution and incorporate a first-order inactivation rate constant in the mobile zone^19,24^, *k*_*MOB*_, yielding ${\psi }_{0}\,(t)$ ~ ${{\rm{e}}}^{-t/\bar{t}}{{\rm{e}}}^{-{k}_{MOB}t}$. We assume that delivery of *Cryptosporidium* to transient storage areas is controlled purely by advective exchange and that gravitational settling is negligible because the Stokes settling velocity of *Cryptosporidium*, dependent on particle diameter size and density, is very low. We define the immobile region as all stream storage areas, including the benthic, hyporheic, and slower-moving surface storage zones. We assume that solutes and pathogenic microorganisms are transported identically in the stream water column owing to the very small settling velocity of viruses, bacteria, and parasites. In this case, hyporheic exchange of solute and *Cryptosporidium* is similar, and *Λ* is characterized by the value for solute hyporheic exchange_._ We assume a power-law residence time distribution within the immobile region^14,18,19,25^ and incorporate a first-order inactivation rate subject to the first order rate for inactivation within the immobile zone, *k*_*IMM*_.

We define the residence time distribution in the immobile region for two scenarios represented by *i* = 1 and 2, where ${\phi }_{1}$ represents pathogens only subject to hyporheic exchange and inactivation in both the mobile and immobile zone and ${\phi }_{2}$ represents all processes included in scenario 1 with the additional pathogen immobilization processes in the immobile zone, such as irreversible filtration, gravitational settling, and attachment and interaction with biofilms and macrophytes. While immobilization is a transient storage process and does not lead to direct removal, we hypothesize that the additional residence time it provides can both (1) enhance the chances for pathogen removal through inactivation in the immobile zone and (2) lead to long-term retention of *Cryptosporidium* that can be resuspended back into the water column during high flow events. For scenario 1, ${\phi }_{1}(t)$ ~ ${t}^{-(\beta +1)}{{\rm{e}}}^{-{k}_{IMM}t}$ or in Laplace space ${\tilde{\phi }}_{1}(u)={[1+{(u+{k}_{IMM})}^{\beta }]}^{-1}\,$within the immobile zone for $0 < \beta  < 1$^19^.

Hydrological storage is often underestimated based on the model choice by assuming that retention timescales have narrow distributions (i.e. exponential), while model fits are improved with a power law residence time distribution of solute in storage^38^. Pathogen residence times are also often underestimated by assuming an exponential residence time distribution of pathogens and microbes within streams. Experimental and theoretical evidence shows that power-law residence times are a more accurate depiction of microbial retention within streams^14,19^. The slow release of pathogens is further supported by the accepted consensus that stream sediments harbor microbes and pathogenic organisms for long times, although this has not been widely incorporated into hydrodynamic models.

The residence time distribution for pathogens in scenario 2,$\,{\phi }_{2}$, describes both the delay in downstream transport that results from pathogens entering the immobile regions via hyporheic exchange, and pathogen immobilization-remobilization within these regions (e.g., from reversible deposition, filtration, and attachment). In Laplace space:

4 $${\tilde{\phi }}_{2}(u)={[1+{[u+{k}_{IMM}+{\Lambda }_{IMM}-{\Lambda }_{IMM}{\tilde{\phi }}_{IMM}(u)]}^{\beta }]}^{-1}$$

where ${{\rm{\Lambda }}}_{IMM}$ is the rate of pathogen immobilization (i.e., immobilization due to filtration, attachment, or deposition) within the immobile region, and ${\tilde{\phi }}_{IMM}(u)\,$is the residence time distribution of pathogens in the immobile zone represented as a power-law distribution with inactivation, ${\phi }_{IMM}(t)$ ~$\,{t}^{-({\beta }_{IMM}+1)}{{\rm{e}}}^{-{k}_{IMM}t}\,\,$ or in Laplace space ${\tilde{\phi }}_{IMM}(u)={[1+{(u+{k}_{IMM})}^{{\beta }_{IMM}}]}^{-1}$, for 0 < *β*_*IMM*_ < 1. Thus, scenario 2 accounts for both pathogen transport into and out of storage areas such as the hyporheic zone and low-velocity surface storage zones, and immobilization and remobilization within these regions. The model equations within scenario 2 have been previously applied to microbial transport in streams^19^.

### Model parameters for reach-scale simulations

Stochastic theory predicts that the slowest mechanism will control the long-term tailing behavior and model parameterization of a tracer concentration vs. time surface water profile^39,40^. This concept can link multiple scales of transport, as previously demonstrated by combining lab (column) scale and field reach-scale studies on solute, particle, and microbial transport and retention in streams^9,19^. We apply this scaling concept to this study by using the column *Cryptosporidium* model parameters from a published study^14^ to characterize the pathogen transport and retention within the immobile zone of the reach scale mobile-immobile model framework. Specifically, *Cryptosporidium* breakthrough curves in a sand column showed power-law behavior^14,15^, with a particle immobilization rate within the immobile zone, ${{\rm{\Lambda }}}_{IMM}$ = 0.2 s^−1^ and a power-law slope of the pathogen residence time distribution within the immobile zone, *β*_IMM_ = 0.35 (Table 1).

**Table 1:** Model input parameters for reach-scale simulations of *Cryptosporidium* inputs to a stream under summer baseflow conditions.

*v*	*D*	Λ	β	Λ_IMM_	β_IMM_	*k*_MOB_	*k*_IMM_
**cm/s**	**m** ^**2**^ **/s**	**1/s**		**1/s**		**1/day**	**1/day**
5	0.095	6 × 10^−2^	0.7	0.2	0.35	0.088	0.011

We assume *Cryptosporidium* release under summer baseflow conditions with an average flow of 60 L/s and an average velocity, *v*, of 5 cm/s. This velocity within an agricultural stream^19^ was associated with a dispersion, *D*, of 0.095 m^2^/s, an exchange rate between the mobile and immobile zone, $\Lambda$, of 6 × 10^−2^ s^−1^, and a power-law slope within the immobile zone, $\beta$ = 0.7. These parameters are reasonable and are within the range of hydrologic model parameters for solute transport within streams during baseflow conditions^25,38^. Inactivation rates of *Cryptosporidium* in the mobile zone (*k*_*MOB*_, surface water) and immobile zone (*k*_*IMM*_, assumed to mainly be the stream sediments) are estimated for summer conditions with water temperatures of approximately 20 °C as 0.088/day and 0.011/day, respectively^41^. A summary of all parameters used within the model simulations is shown in Table 1. The model simulations were run with the 1-month scenario detailed in the previous section. The 1-month duration of the release was chosen as an arbitrary reference duration to assess the permanence of oocysts in the stream even after the release has stopped.

A total count of *Cryptosporidium* oocysts immobilized and inactivated were determined at each sampling distance and at different timepoints of interest (i.e., 1 month to 2 years). A model output breakthrough curve (number of oocysts vs time) with and without inactivation was produced. These model outputs were integrated to the different timepoints of interest using the trapezoidal method to determine a total number of oocysts that passed by the sampling point within the surface water with and without inactivation. The difference between with and without inactivation was used to calculate the number of oocysts inactivated vs. immobilized within the stream at each timepoint of interest. The # of oocysts immobilized at each downstream distance was estimated as the difference between the previous sampling point (i.e., the value at 300 m represents the reach between 100–300 m and is taken as the difference between the cumulative sum of oocysts at 300 m and the cumulative sum of oocysts at 100 m). The values for % *Cryptosporidium* immobilized were calculated by dividing the total # immobilized by the known model input of *Cryptosporidium* oocysts (i.e., a 1-month input).

## Results

### Transmission of Cryptosporidium in stream surface water

**Figure 2:**
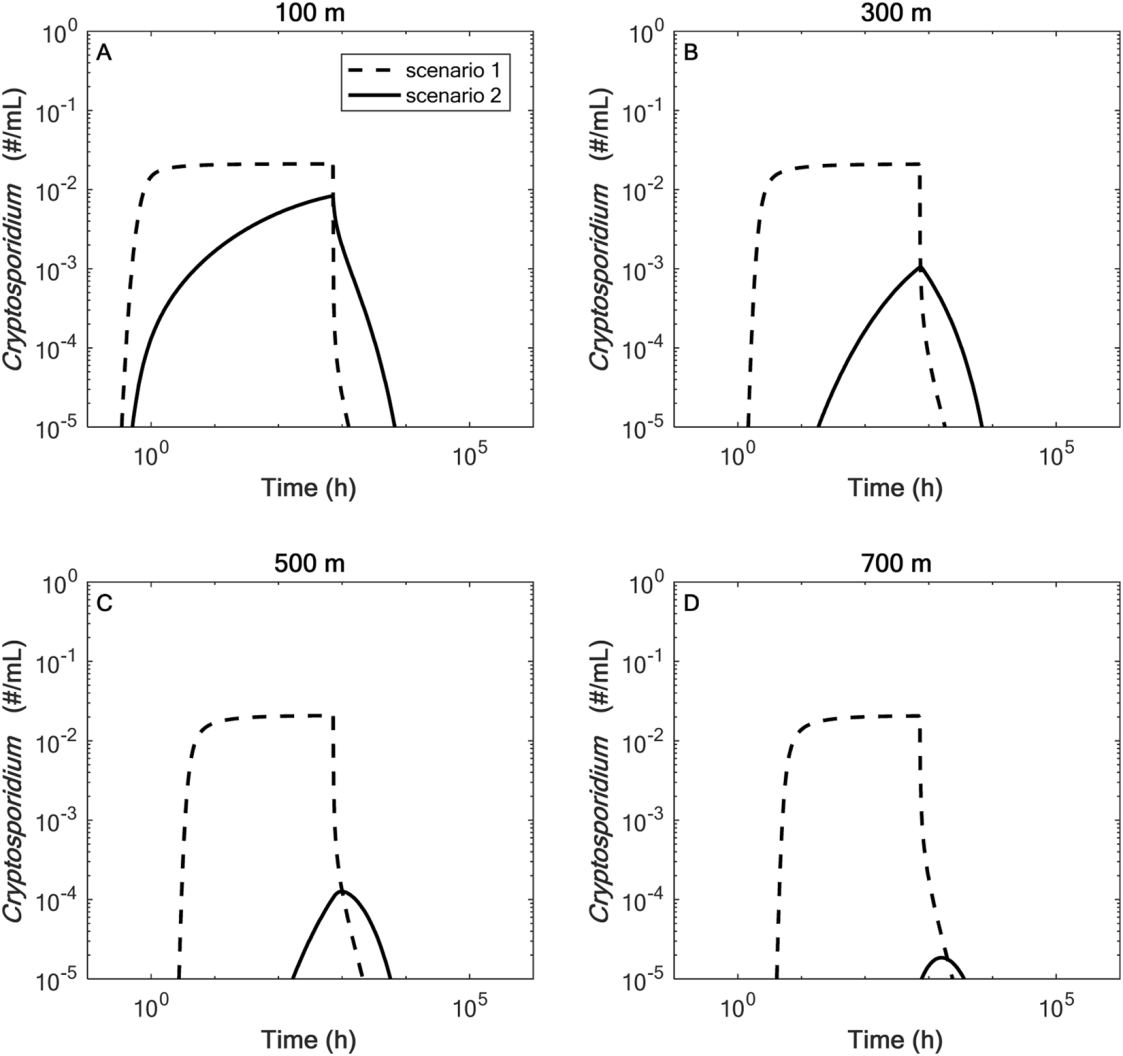
In-stream breakthrough curves of *Cryptosporidium* in the mobile (in-stream) zones at (**A**) 100, (**B**) 300, (**C**) 500 and (**D**) 700 m from the input for scenario 1 (black slotted lines) and scenario 2 (black solid lines). Scenario 1 shows *Cryptosporidium* with in-stream transport, inactivation and hyporheic exchange, but without the immobilization processes (e.g., reversible filtration), while scenario 2 shows *Cryptosporidium* with immobilization processes that increase retention.

Model simulations for a 1-month input of *Cryptosporidium* to an agricultural stream show in-stream pathogen counts at 100, 300, 500, and 700 m downstream of the input (Fig. 2). *Cryptosporidium* transmission is presented under two scenarios (1) with only hyporheic exchange and inactivation (black slotted lines, Fig. 2) and (2) with hyporheic exchange, inactivation, and additional immobilization processes in transient storage areas (black solid lines, Fig. 2). For scenario 1, a decrease in maximum in-stream concentrations from 2.1 × 10^−2^ #/mL at 100 m to 2.0 × 10^−2^ #/mL at 700 m downstream of the input demonstrates how hyporheic exchange delays downstream transport, but does not greatly reduce the maximum in-stream concentration. As described previously^34^, a safe water supply is considered to have less than 10^−5^ oocysts/mL. This value assumes a typical human consumption of 2 L/day and a safety/error factor of 300 to 1,000, which is typical for public health standards. In-stream concentrations remained above 10^−5^ oocysts/mL for 1269 and 2357 hours for sites 100 and 700 m downstream of the input, respectively. When immobilization processes are considered retention is increased to an even greater extent and peak in-stream concentrations range from approximately 8.1 × 10^−3^ to 1.8 × 10^−5^ oocysts/mL at 100 m and 700 m, respectively. Thus, oocyst in-stream concentrations quickly decreased when immobilization processes were considered, which can then remain immobilized within the sediments and remobilize at a later time (Sec. 4.2).

### Long-term persistence of Cryptosporidium under baseflow conditions

**Figure 3:**
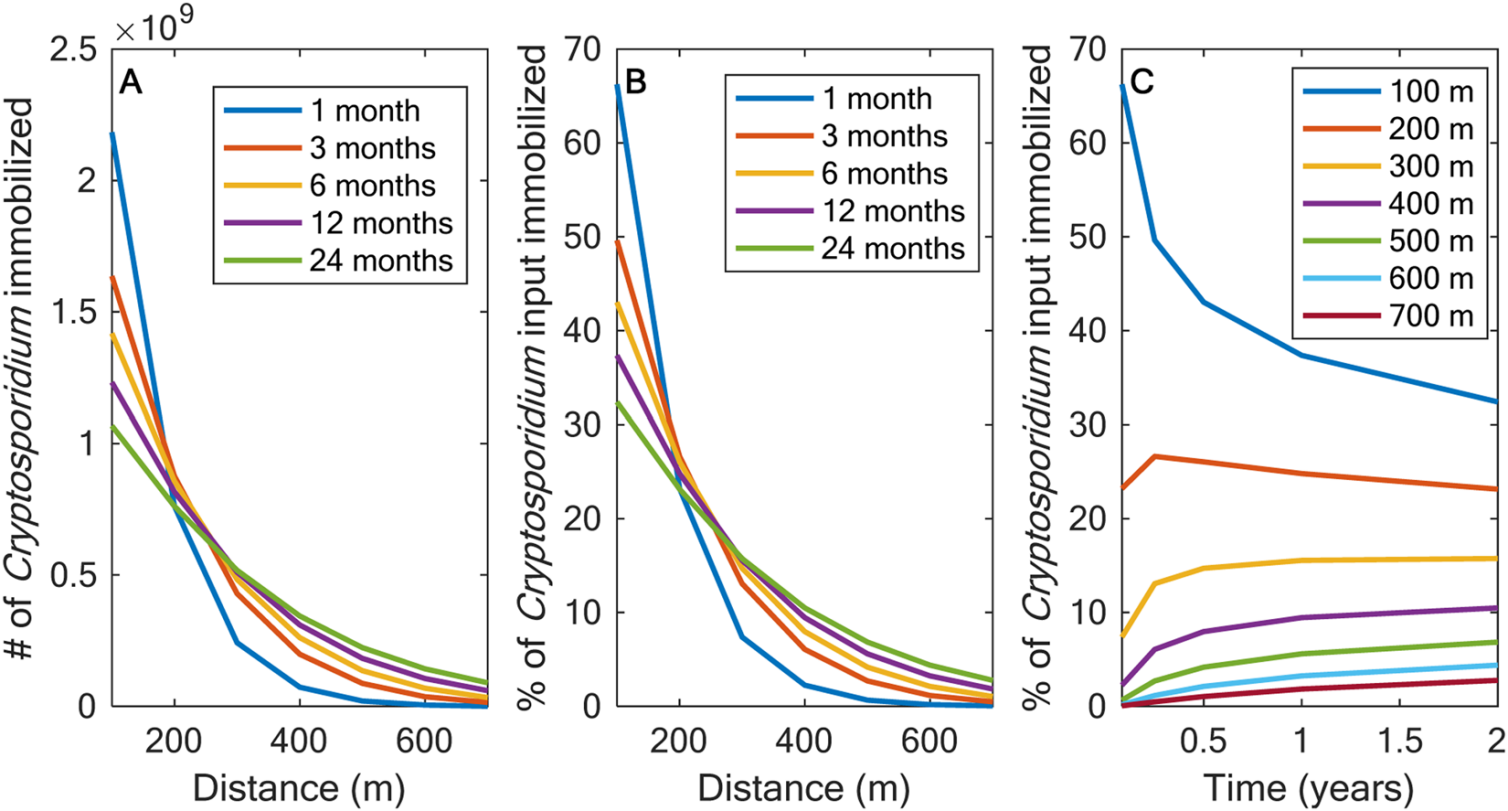
*Cryptosporidium* immobilized within the sediments and other transient storage areas during a 1-month input. Longitudinal profile of *Cryptosporidium* (%) immobilized at multiple times after a 1-month input at multiple times (**A**) and *Cryptosporidium*, represented as % of input (**B**) and total # of oocysts (**C**) that remains within the stream reach (i.e. 100–700 m from input) over time.

In scenario 1, 99.7% of the oocyst inputs already passed 100 m at 1 month with only 0.1% of the inputs inactivated, however when immobilization processes were considered only 33.8% were observed in the mobile phase (66.2% immobilized and 1.4% inactivated) prior to the stop of the input (Fig. 3). Although in-stream concentrations decrease relatively quickly (within hours), pathogen accumulation within immobile zones (i.e., sediments and slower moving surface water) results in high counts within these areas for long periods of time (i.e., months to years) (Fig. 3). After relatively quick immobilization, *Cryptosporidium* is slowly released back to the water column, where at 100 m the *Cryptosporidium* immobilized decreases from 2.2 × 10^9^ to 1.4 × 10^9^ oocysts, 66.2% to 43.0% of the inputs, from 1 to 6 months (Fig. 3A,B). These results demonstrate that *Cryptosporidium* persists for months at 100 m and may rework its way downstream, but will remain within the first 700 m for years after the input under baseflow conditions (Fig. 3).

## Discussion and Conclusions

When considering a pathogen with such a low infectious dose, the retention within streambeds can dramatically increase the time during which thresholds for safe consumption are exceeded. These results demonstrate that both hyporheic exchange and immobilization processes within sediments and other slower moving areas increase pathogen persistence in streams.

*Cryptosporidium* oocysts are readily immobilized within the first 100 m (~66%, Fig. 3) and stay for days to years within transient storage areas. In fact, at 100 m *Cryptosporidium* in-stream levels do not reduce to below 10^−5^ oocysts/mL until 280 days after the start of a 1-month input. At 700 m from the simulated input of *Cryptosporidium*, in-stream concentrations only slightly exceeded 10^−5^ oocysts/mL for a short period of time, but the majority were immobilized in transient storage areas within the 700 m reach downstream of the source. The numbers of viable oocysts remaining in the 100 m reach downstream of the input decreased over time, while the numbers of viable oocysts from 100–300 m increased and then decreased from 0 to 24 months and the reaches further downstream showed a consistent increase in the number of viable oocysts. The constant reworking of sediment beds has previously been observed^18,36,42,43^, and is a combination of flushing, trapping, and accumulation of fine sediment and microbes that occurs both at baseflow and during high flow events. The model scenario was for 1 month and only one stream inlet, but the landscape is continuously adding *Cryptosporidium* to the stream, which can result in even more accumulated pathogens within the stream. As non-point sources are distributed among the watershed, there will be constant and sporadic inputs simultaneously, but the majority of the input will be deposited within the first 100 m, at least initially before remobilization processes move the pathogenic microorganisms further downstream.

Cryptosporidiosis remains an important waterborne disease in both the United States and Europe, posing significant public health and economic problems^44–47^. Likely the high risk is due to a combination of a low consistent input of *Cryptosporidium*, long retention times, and low inactivation rates that can result in long-term accumulation within streams. Accumulated pathogens can subsequently resuspend slowly back into the water column during baseflow or as a pulse in response to a storm event. Previous cryptosporidiosis outbreaks have occurred following a high flow event^48,49^, where microbes are rapidly resuspended due to scour of streambed sediments. The extent of microbial resuspension is dependent on the frequency of high flow events, microbial colonization of benthic biofilms, macrophytes, and sediments, and potential for erosion or remobilization from these reservoirs^50^. Implications of pathogen persistence within stream storage areas, and in particular sediments, includes increased risk especially when climate change is expected to increase the duration of drought periods and lead to intensified rain events. This model framework can be used to help predict response to storm events as pathogens remobilized during high flow conditions are directly related to the in-stream source of pathogens accumulated during baseflow^20–23^. Furthermore, this model framework can incorporate new information to improve predictions of specific *Cryptosporidium* species. The use of molecular diagnostic tools has significantly improved our understanding of cryptosporidiosis epidemiology^51^ and now it is known that human cryptosporidiosis is predominantly caused by *C. hominis* and *C. parvum*. It has also recently been discovered that *Cryptosporidium* can multiply without host cell encapsulation (in host cell-free systems such as aquatic biofilms), which potentially poses an even greater environmental risk^52–54^. Overall net inactivation was observed in our study case, but specific sub groups and growth can be incorporated into the model framework as needed for specific cases or applied to other pathogenic bacteria of interest. This could be especially useful if supporting lab-scale experiments have been conducted to parameterize the retention within the sediments, previously shown to control the late-time tailing of observed in-stream breakthrough curves^19^.

Here we presented how the mobile-immobile model framework is a first step for accurately characterizing pathogen transport and retention in stream. A mobile-immobile framework can accurately characterize pathogen dynamics in streams and incorporate key processes that lead to long residence times. The fate of the pathogens after entering a stream and the long-term retention has been underestimated if hyporheic exchange and immobilization processes within sediments and other storage areas is not considered. The multiscale model enables information on pathogen retention in column experiments to be related to net downstream transport at the stream-reach scale. Therefore, while it is not possible to conduct field scale tracer experiments with pathogens, lab scale studies, such as column experiments can be used to parameterize reach-scale estimates, as demonstrated within this modeling study. The combination of stream reach-scale analysis and multi-scale modeling improves assessment of *Cryptosporidium* transport and retention in streams to predict downstream exposure to human communities, wildlife, and livestock. The mobile-immobile model framework can be modified to any system of interest to estimate baseflow pathogen accumulation and therefore help predict the potential loads of resuspended pathogens from the streambed to the water column in response to a storm event.

### Data Availability

The MATLAB source code used to generate the model simulations is provided online at 10.6084/m9.figshare.5756304.
